# Supplementary material for: The effectiveness and cost of integrating pharmacists within general practice to optimize prescribing and health outcomes in primary care patients with polypharmacy: a systematic review
Source: BMC Prim Care. 2023 Feb 6;24:41. doi: 10.1186/s12875-022-01952-z (PMC9901090; doi:10.1186/s12875-022-01952-z)
Supplement: Supplementary file 2 — Additional file 2. Domains of integration; summary table [file 12875_2022_1952_MOESM2_ESM.docx]

# Additional file 2

## Domains of integration

| **Study** | **Organisational** | **Informational** | **Clinical** | **Functional** | **Normative** | **Financial** |
| --- | --- | --- | --- | --- | --- | --- |
| **Carter** | ✓ | ✓ | ✓ |  |  | ✓ |
| **Hanlon** | ✓ | ✓ | ✓ |  |  | ✓ |
| **Sellors** | ✓ | ✓ | ✓ |  |  | ✓ |
| **Taylor** | ✓ | ✓ | ✓ |  |  | ✓ |
| **Geurts** | ✓ | ✓ | ✓ |  | ✓ |  |
| **Britton** | ✓ | ✓ | ✓ |  |  |  |
| **Campins** | ✓ | ✓ | ✓ |  |  |  |
| **Graffen** | ✓ | ✓ | ✓ |  |  |  |
| **Granas** | ✓ | ✓ | ✓ |  |  |  |
| **Jameson** | ✓ | ✓ | ✓ |  |  |  |
| **Krska** | ✓ | ✓ | ✓ |  |  |  |
| **Leendertse** | ✓ | ✓ | ✓ |  |  |  |
| **Lenaghan** | ✓ | ✓ | ✓ |  |  |  |
| **Sloeserwij** | ✓ | ✓ | ✓ |  |  |  |
| **Van der Meer** | ✓ | ✓ | ✓ |  |  |  |
| **Verdoorn** | ✓ | ✓ | ✓ |  |  |  |
| **Zillich** | ✓ | ✓ | ✓ |  |  |  |
| **Sorensen** | ✓ |  | ✓ | ✓ |  |  |
| **Bryant** |  | ✓ | ✓ |  |  |  |
| **Kwint** |  | ✓ | ✓ |  |  |  |
| **Vinks** |  | ✓ | ✓ |  |  |  |
| **Varas-Doval** | ✓ |  | ✓ |  |  |  |
| **Bernsten** |  |  | ✓ |  |  |  |

Text highlighted in bold indicates a heading.
